# Effect of Design Modification on the Distortion Behavior of a Complex Counter Gear

**DOI:** 10.3390/ma14082022

**Published:** 2021-04-17

**Authors:** Jwalant Kagathara, Thomas Lübben, Matthias Steinbacher

**Affiliations:** 1 Leibniz Institute for Materials Engineering—IWT, Badgasteiner Straße 3, 28359 Bremen, Germany; Luebben@iwt-bremen.de (T.L.); steinbacher@iwt-bremen.de (M.S.); 2 MAPEX Center for Materials and Processes, University of Bremen, 28359 Bremen, Germany

**Keywords:** numerical simulation, distortion, lightweight construction, counter gear, case hardening, SAE 5120 (20MnCr5)

## Abstract

A change in component design could help achieve objectives in lightweight construction. However, lightweight component design can incur serious distortion problems after the final heat treatment due to reduced stiffness or asymmetries in the mass distribution. The analysis of design modification through geometrical variations and their consequences on the distortion behavior through experiments can be costly and time consuming. In this paper, using 3D simulation models, different modified lightweight geometries are simulated. Using these simulation results, the authors try to understand the complex distortion behavior and correlate it with the effects of design modification.

## 1. Introduction

In recent years, weight reduction has been the prime goal in automobile industries to cater for the needs of economic and ecological aspects. Apart from the application of new materials with considerably lower density, a change of component design can be used to succeed in aims regarding lightweight construction.

The common design approach to acheive reduced weight is the manufacturing of gears with thin bodies, possibly with holes and slots. Shweiki et al. [1] analyzed the impact of two different gear weight reduction strategies, decreasing the web thickness, and material removal through holes or slots, on the dynamic behavior of gears. In [2], the authors used a cellular lattice structure gear body, which reduced the weight of the gear as well as reduced the vibrations. However, the studies do not consider a production-oriented approach during the design process. The gears are usually heat-treated after the forming process to improve the existing structural and application properties of the material. The lightweight design and construction of the gears lead to reduced stiffness and asymmetrical mass distribution, which induces serious distortion problems after the final heat treatment.

It is of utmost importance to obtain knowledge of the basic mechanisms leading to dimensional and shape changes because of heat treatment to improve the design and reduce the post-processing machining cost and time. This analysis can be pursued either using suitable in situ measurement techniques or using qualified simulation software. However, the first approach is usually not applicable in heat treatment facilities, or only to a minimal extent, despite the greatest effort. Furthermore, in many cases, only the final state can usually be investigated in experimental studies, thus lengthy and cost-intensive test series have to be carried out. The simulation of heat treatment processes, on the other hand, is free of such constraints and can be very helpful to identify important variables that cause dimensional and shape changes and to understand the associated processes.

The continuous development of heat treatment simulation over the last few decades has made it possible to simulate and calculate complex geometries such as sliding sleeves [3] or gears [4,5,6,7]. Due to the still considerable computational time required to simulate such components, reduced models with the consideration of periodic boundary conditions or 2D models are usually considered. Furthermore, the complex interactions between material properties and process parameters sometimes make it difficult to precisely predict size and shape changes. Provided that the material behavior [8,9] (for example, transformation behavior) and process parameters (for example, heat transfer [10]) are modeled correctly, good agreement between experiment and calculation can be achieved.

The heat treatment simulation is preferably focused on the quenching processes of quenched and tempered steels and rolling bearing steels and the carburizing and quenching of case-hardened steels [11,12,13,14]. In the last decade, the effect of segregation on distortion behavior has been extensively researched using both experiments and simulation [15,16,17]. Furthermore, in recent times, process chain simulations have gained importance, and work has been carried out on the combination of forging and heat treatment with a focus on segregation [18], as well as forming, heat treatment, and crash [19]. However, most of the research focuses on improving and optimizing processes and process steps, understanding the influence of different effects arising during heat treatment on final properties and distortion, and the effect of different manufacturing processes on the final distortion behavior. However, most of the studies use single-piece geometry for their research and analysis.

There exist very few systematic studies of effects by design modifications on the final distortion behavior. In [4,20], the authors developed the first design rules for gear-based bodies that considered the distortion potential into account and led to components with tolerable size and shape changes. However, the effects of gear teeth on the overall distortion behavior were not considered in the study. The authors in [21] showed that the influence of the gear tooth has to be taken into account for the size and shape change of a gear rim, and also for the tilting of the web, gear rim, and gear tooth. However, this distortion behavior is specific to the gear geometry chosen by the authors in their study. Recently, in [22], the authors showed case hardening experiments using the example of a weight-reduced counter gear made of SAE 5120 (20MnCr5). The experimental results showed significantly different distortion behavior depending on the geometry and process parameters. As mentioned, analysis using only experiments can be a cumbersome task, and in this regard, simulations can be constructive for understanding this complex distortion behavior.

In this paper, 3D simulation models are developed for different modified counter gear geometries, and heat treatment carburizing simulations are performed. The simulation results are validated with those of experimental results, and different distortion criteria are analyzed. Furthermore, using these simulation results, the authors try to understand the complex distortion behavior and correlate it with the effects resulting from design modification. The results achieved here will be the basis for future compensation measures and the optimization of cross-sectional areas of the gear to study their effect on distortion behavior.

## 2. Material and Methods

### 2.1. Material

The case-hardened steel 20MnCr5 (EN10084-2008) was used for the investigations, and the chemical composition is given in Table 1. The composition complies with the standard specification.

In this study, to better characterize the material regarding phase transformation by dilatometry, instead of a change of length, the volume change was considered. Therefore, two samples each from the axial and radial direction were investigated. Furthermore, carburized dilatometer samples (0.21 ma.% C and 0.289 ma.% C) were examined to determine the carbon dependence of the phase transformation for the investigated melt of 20MnCr5. The dilatometer tests were performed with constant austenitizing conditions (3 min, 880 °C, 20 min) and different cooling rates to derive the parameters for the transformation models and thermal strain during austenitizing and quenching.

### 2.2. Experimental Procedure

The work is carried out in a close coupling of experimental and numerical investigations. On the one hand, experimental results served to obtain a database on the distortion behavior of the various geometry variants and heat treatment processes (see more details in [22]), while on the other hand, they were also used to validate the simulation. For the investigations, the case hardening steel SAE 5120 (20MnCr5) was used.

**Figure 1:**
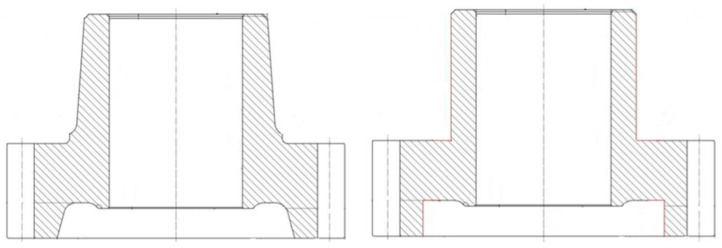
Original counter gear (**left**) and simplified basic geometry (**right**).

**Figure 2:**
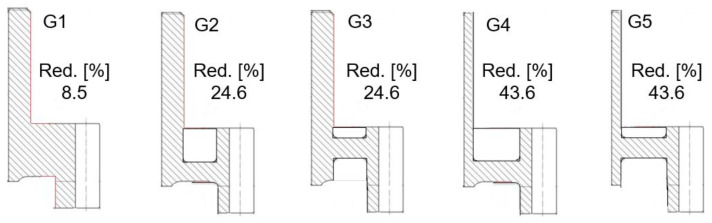
Geometry variations G1–G5 and percentage of mass reduction achieved.

The original counter gear of the normal module 4.32 has 31 teeth and a mass of 6.1 kg. The gear has a tooth tip diameter of 162 mm and a width of 107 mm. In the first step, the original geometry of the counter gear was simplified by straightening the chamfers of the outer hub surface and the gear rim in the lower area, Figure 1. The motivation for these simplifications is a simplified interpretation of the expected form changes and an increased susceptibility to form changes. The geometry variant G1 (simplified geometry) thus has the maximum possible widths of the hub and gear rim. For further mass reduction, the hub width, the web width, and the gear rim width were varied. Additionally, the web position was changed to reduce the installation space. Thus, variants G2 and G3 are defined by a reduced web width and G4 and G5 by reduced web and hub widths. G2 and G4 have a practically symmetrical web position in relation to the gear rim, while G1, G3, and G5 have asymmetrical web positions. The geometries G1–G5 and the achieved mass reduction with respect to the original geometry are shown in Figure 2.

The standard case hardening of the five geometry variants was carried out in an Aichelin W3 chamber furnace (AICHELIN Holding GmbH, Mödling, Austria) using gas carburizing and subsequent oil quenching. The carburizing temperature was 920 °C, and quenching was carried out from 870 °C in an agitated W72 high-speed quenching oil bath at 60°C and maximum possible circulation. A detailed description of the experimental investigation can be found in [22] concerning the manufacturing process of the gear, the heat treatment, the distortion measurement by the 3D coordinate measurement machine, the measurement positions, and the determination of the size and shape changes.

The temperature measurement experiments were conducted to derive the location and temperature-dependent heat transfer coefficient (HTC) for simulations. The temperature was measured during oil quenching at 10 different positions. The positions were chosen so that at least one measuring point near all relevant surfaces forming the outer contour of the component was represented. The temperature measurement experiments were conducted on the two extreme geometry variants G1 and G4, to include the influence of the component geometry on the HTC.

### 2.3. Simulation Procedure

**Figure 3:**
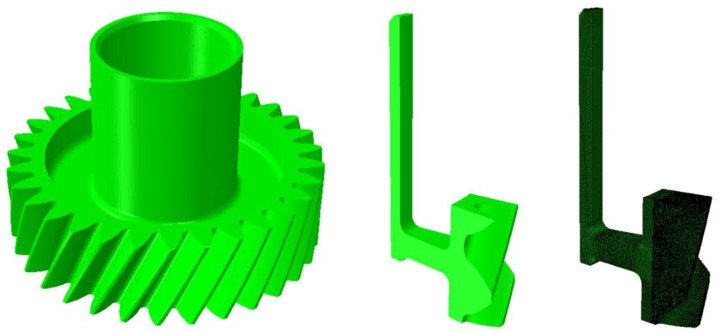
A 3D model of the weight reduced gear (**left picture**), one tooth model (**middle picture**), and the one tooth meshed model (**right picture**) [23].

An example of the 3D one tooth model of the geometry type G4 used for the simulation is shown in Figure 3. Due to the complicated tooth geometry, two different mesh types were applied for all geometry types. The tooth region was meshed with a tetrahedral mesh, while the gear body was meshed with hexagonal elements. For the coupled thermal–mechanical calculation, elements of the type C3D8T and C3D4T were used. The geometry model for the diffusion calculation (carburization) consisted of elements of the types DC3D8 and DC3D4. The number of elements was approximately 434,000, and the simulation time was around 5 days. In the simulation, the cyclic boundary condition had to be considered when using the one tooth model. Therefore, the geometry was meshed with matching meshes on the surfaces in the tangential direction in ABAQUS^®^ 6.14-2 in such a way that the nodes of the left and right surfaces were at equivalent positions.

The simulation of the heat treatment process of the modified counter gears included austenitization, gas carburization, and oil quenching. At first, a fully coupled thermo–mechanical analysis of the austenitization was performed to create the temperature profile necessary for the carbon diffusion analysis. In the next step, a gas carburization process was simulated. During transport into the quenching chamber, only heat radiation was taken into account, as the convective heat transfer was comparatively small and could therefore be neglected. The initial conditions for the quenching process simulation corresponded to the distributions of temperature, phase fractions, and carbon content from the previous calculation steps. The boundary conditions for the quenching process were based on the experimental work and consisted of the local and temperature-dependent heat transfer coefficients and the temperature of the quenching medium. Apart from these, the following temperature, phase, and carbon-dependent material properties and process boundary conditions are included in heat treatment simulation:

- phase transformation during heating and quenching
- transformation-induced plasticity (TRIP) during heating and quenching
- creep during heating
- yielding with isotropic hardening during quenching
- carbon diffusion during gas carburizing

A user-defined subroutine is implemented into the commercial finite element analysis (FEA) package ABAQUS^®^ regarding all relevant material effects, including thermal dilatation, phase transformation dilatation, classical plasticity, transformation-induced plasticity, and creep of the steel grade SAE 5120 (EN 20MnCr5) [8,9]. The parameters for the transformation plasticity and mechanical behavior of the steel were taken from the dataset developed within the Collaborative Research Center “Distortion Engineering” [24,25]. However, the important, strongly melt-dependent parameters such as the transformation behavior, thermal expansion, and anisotropic transformation strain, if any, for the melt used in this study were re-determined by dilatometry (Baehr 805 A/D, TA Instruments, Hüllhorst, Germany).

**Figure 4:**
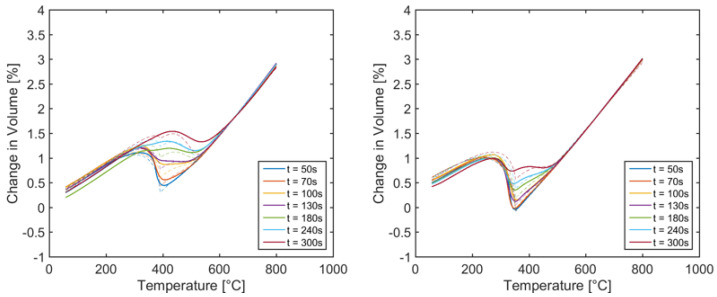
Comparison of the results from the simulation (**dash lines**) and the experiment (**solid lines**) for the elongation behavior as a function of cooling time and C-content (**left**: 0.21%, **right**: 0.281%) for the compromise model [23].

**Figure 5:**
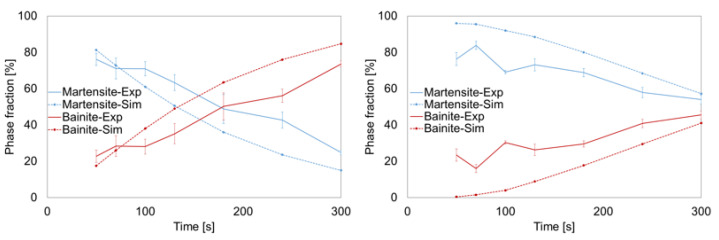
Comparison of the results from the simulation and the experiment of phase fraction as a function of cooling time and C-content (**left**: 0.21%, **right**: 0.281%) for the compromise model [23].

The transformation model parameters were adjusted in several iterations based on the dilatometer tests and resulting metallographic results. Despite various modifications on the model level, no model data set could be determined to describe both the transformation kinetics and the description of the resulting microstructure concerning the phase fractions. Since both the transformation kinetics via the mechanisms of transformation plasticity and the phase fractions via their specific density exert effects on the shape and size changes, a compromise model was developed concerning the agreement between these two criteria. The comparisons between experiment and simulation regarding the change in volume and the resulting phase fractions are documented in Figure 4 and Figure 5.

With regard to the transformation kinetics and the change in volume, the differences between the experiment and the simulation occured primarily in the area of bainite transformation. The comparison of the results concerning the phase fractions revealed significant differences for the range of the quenching time from 50 s to 130 s for 0.289 ma.% C-content, whereas it was the other way round for 0.21 ma.% C-content (Figure 5).

### 2.4. Heat Transfer Coefficient

The heat transfer coefficient (HTC) was determined for the 3D model for the geometry types G1 and G4. In order to have a realistic starting point for the adaptation of the simulation to the measurements from the trial and error procedure, a master curve defined by only five points and roughly reproducing the original measurement curve was used [4]. The values of the HTC at the five points of the master curve were varied for each surface segment through trial and error procedures until a minimal difference between measurement and calculation resulted at all measuring positions during the entire quenching process. The detailed description is documented in [23].

**Figure 6:**
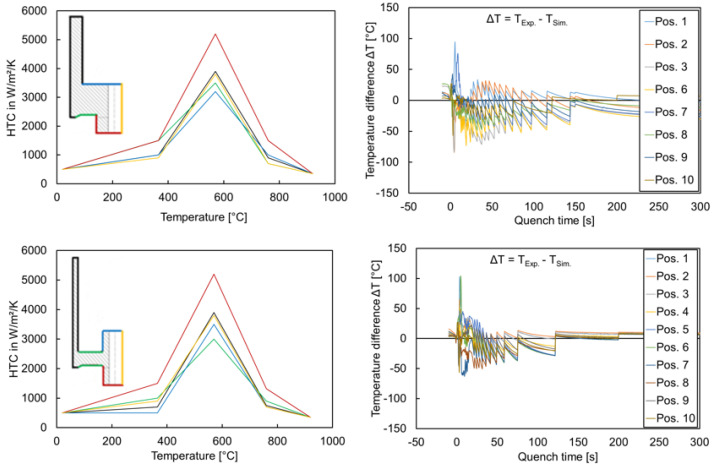
Adjusted HTC and local temperature differences between the measured and calculated temperature curves during transport and oil quenching for geometry types G1 (**top**) and G4 (**bottom**) [23].

A good agreement between the calculated and measured temperature curves could be achieved. Apart from short exceptions at the beginning of the quenching, a maximum temperature difference of around 60 °C is obtained for all temperature measuring points (Figure 6).

The heat transfer coefficient was not possible to adjust for geometry types G2, G3, and G5 due to unavailable temperature measurement data. It was initially assumed that the location-dependency of HTC would not have a significant influence, as was found in the previous project [4]. However, the geometry-dependency of HTC indeed influenced the tilting behavior [23]. Therefore, different attempts were made to estimate the HTC distributions for G2, G3, and G5 from the HTC distributions of G1 and G4. The geometry G2 was assumed to have a similar temperature distribution to G1 due to the thick hub. The geometry G3 was also assumed to have the same HTC as G1; however, the shape and size change results for geometry G3 were not satisfactory. The reason could be the position of the web. Due to the upmost possible web position, the cooling at the lower web and gear rim areas could be slower than in geometry type G1. Therefore, the HTC at the bottom of the web and gear rim was further modified, i.e., the value was chosen lesser than the values adjusted for G1. Analogously, the assumption and modification were performed for geometry G5 based on the temperature measurement results of G4. Table 2 summarizes these assumptions.

**Figure 7:**
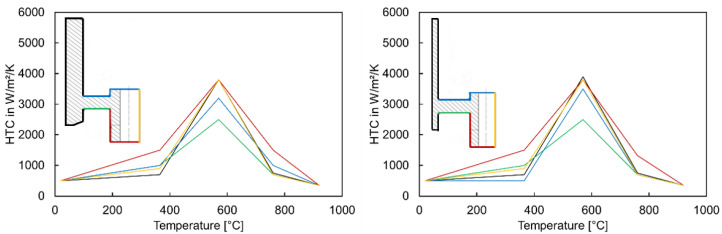
Adjusted HTC for geometry types G3 (**left**) and G5 (**right**).

Due to unavailable experimental data regarding temperature measurement, comparisons of the tilt angles between the simulation and the experiment were considered as criteria for adjusting the HTC of geometry types G2, G3, and G5. The values of the HTC at the web bottom and gear rim were varied through the trial and error procedures until a satisfactory agreement for tilt angle was achieved. The adjusted HTC for the geometry types G3 and G5 are shown in Figure 7.

## 3. Results and Discussions

The 3D simulation models were developed for all the investigated geometry variations (G1, G2, G3, G4, and G5), and carburizing simulations were performed. The size and shape changes due to heat treatment were analyzed based on four criteria: change in radius at the outer hub, change in radius at the inner hub, change in radius at the outer gear tooth, and change in height at the web and gear tooth. Apart from size and shape changes, gear tooth tilting in the radial direction and tilting of the web and gear rim in the axial direction was also compared and analyzed. Furthermore, the simulation results were also validated concerning hardness and phase fraction. The calculated values for phase fractions concerning austenite, martensite, and bainite, and hardness values at three different positions show good agreement with the experimental values. The detailed validation is documented in [23].

### 3.1. Size and Shape Changes

**Figure 8:**
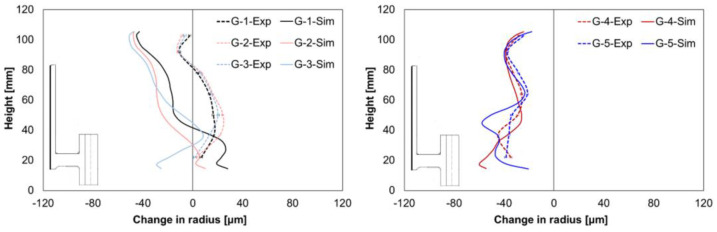
Comparison of measured and calculated radius changes at the hub inside for G1–G3 (**left**) and G4–G5 (**right**).

The comparison of the experimental and simulated values for the change in radius at the inner hub area shows different mean size changes for geometry types G1–G3, but a nearly perfect match for the results from geometry types G4 and G5, Figure 8. In the experiments, a nearly positive alteration for the geometry types G1–G3 was observed, whereas contractions were found in the simulation. However, the simulation results show almost correct trends for shape changes.

**Figure 9:**
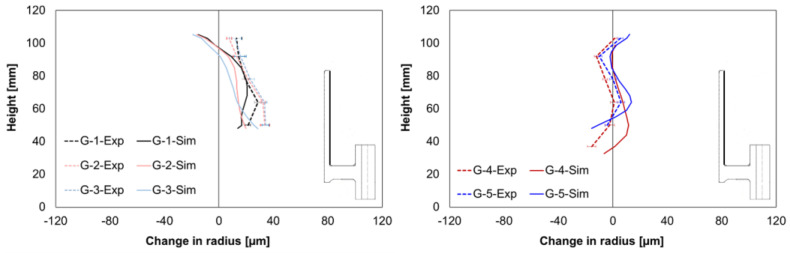
Comparison of the measured and calculated radius changes at the hub outside for G1–G3 (**left**) and G4–G5 (**right**).

A good agreement between the simulation and experimental size change values was achieved regarding the outer hub area (Figure 9). In addition, for the geometry types G4 and G5, the simulation model reproduced correct shape change, whereas it differed for geometry types G1, G2, and G3.

**Figure 10:**
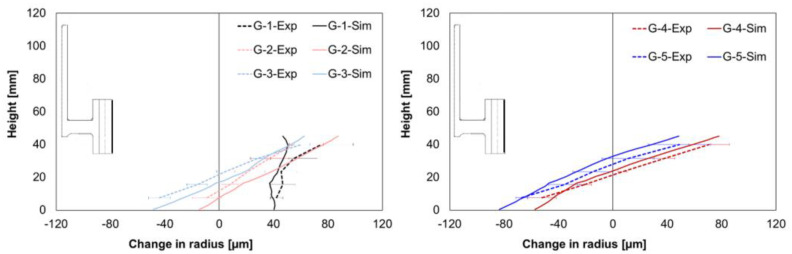
Comparison of the measured and calculated radius changes at the gear tooth for G1–G3 (**left**) and G4–G5 (**right**).

The simulated change in the radius at the outer gear tooth area is illustrated and compared for all the geometry types in Figure 10. The mean size changes from the simulation differed from the experimental values but were within the standard deviations, except for geometry type G3. In addition, the simulation results showed the correct trends for the shape changes for all geometries.

**Figure 11:**
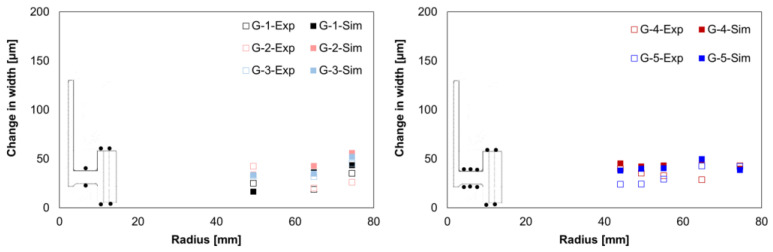
Comparison of the measured and calculated width changes for G1–G3 (**left**) and G4–G5 (**right**).

Regarding the local width, the comparison of the experimental and simulated values showed considerable mean size changes, Figure 11. Furthermore, as in the experiments, positive alterations were found in the simulation results. The simulated values were slightly lower for the web area and slightly higher for the gear rim area than the experimentally determined values.

### 3.2. Tilting Behavior

**Figure 12:**
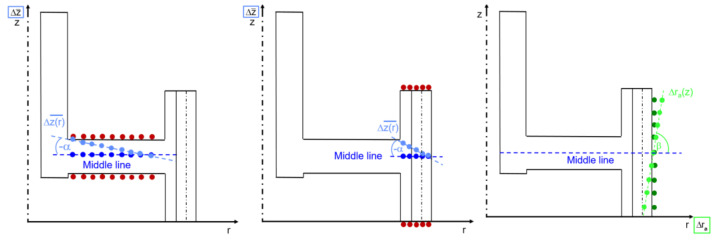
Calculation of the tilt angle at the web (**left**), gear rim (**middle**), and gear tooth (**right**).

In accordance with the procedure for evaluating the experimental investigations of the gear, the focus of the numerical distortion analysis was also placed on the tilting of the gear wheel in the axial direction and the gear tooth in the radial direction. In [23], the authors showed that there is no linear behavior over the entire area of the web and gear rim in the axial direction. Therefore, the tilting of the web and the tilting of the gear rim in the axial direction were considered as separate distortion criteria, and an additional tilting angle was introduced (Figure 12). Furthermore, from Figure 10, it can be seen that the change in radius was not exactly linear. Therefore, the tilting of the gear tooth in the radial direction is described using a parabolic function. In contrast, the tilting of web and gear rim/tooth in the axial direction (Figure 11) can be approximated using a linear function.

**Figure 13:**
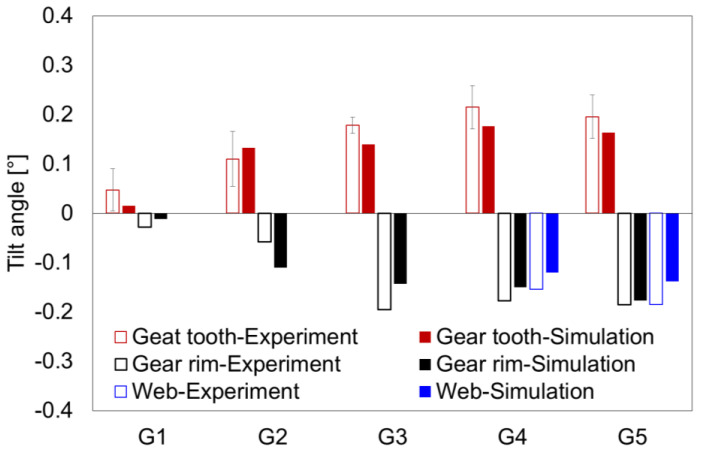
Comparison of the experimentally determined and calculated tilt angles of the gear tooth, gear rim, and web for geometry types G1 to G5.

The simulated tilt angles of the gear tooth, gear rim, and web (if more than one measuring point was present in the area) are shown and compared with the experimental values in Figure 13. The simulation model calculated good results for all geometry types. Significantly, the simulation model correctly predicted the direction of the tilt. In detail, the gear tooth tilt angle calculated from the simulations lay within the error limit, except for geometry type G3. In the experiments, the change of *z*-coordinate at the web bottom was measured for only one component per type of geometry, and therefore, the calculation of standard deviations was not possible. However, the differences between the experimentally determined and calculated tilt angles were still acceptable.

### 3.3. Understanding Tilting Behavior with Regard to Design Modification

In the previous study [23], the authors performed a numerical investigation using the example of geometry type G4 with and without a gear tooth to identify the underlying mechanisms for the tilting and to understand the causes. The study started with very strong simplifications and systematically approached reality. The results showed that the tilting of the gear tooth in the radial direction and the tilting of the web and gear rim in the axial direction result from the interplay of the different asymmetries of mass distribution, temperature distribution, and distribution of the carbon content.

In this study, the focus is on the design modification at different areas of the gear and its implication on distortion behavior. The analysis is concentrated on the tilting behavior at the gear tooth, gear rim, and web. Nevertheless, in the experimental work, it was not possible for some geometry types to analyze the tilting of the web due to insufficient measuring points, but it is included in the simulation work.

#### 3.3.1. Basic Geometry

**Figure 14:**
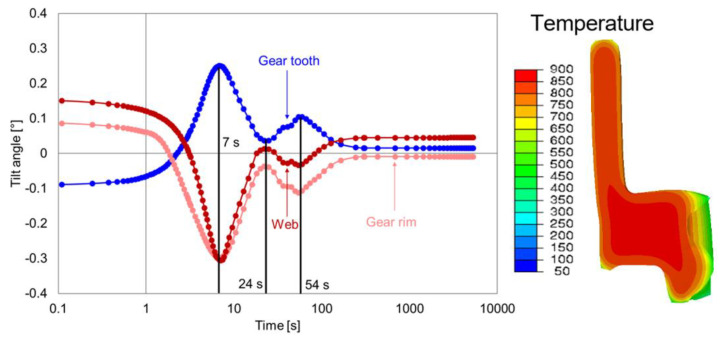
Tilt angle development of the web, gear rim, and gear tooth for the geometry type G1 (**left**), and a contour plot with temperature distribution at 2 s (**right**).

The geometry G1 had the maximum possible width of the hub and web. The development of the tilt angles of the gear tooth, gear rim, and web during quenching is shown in Figure 14. The effect of heating and carburizing was clearly noticeable, as the tilt angle was large at the start of the quenching process. During heating, the thermal, creep, and transformation strains were responsible for tilting behavior, whereas the carbon infusion during carburizing influenced the tilting.

**Figure 15:**
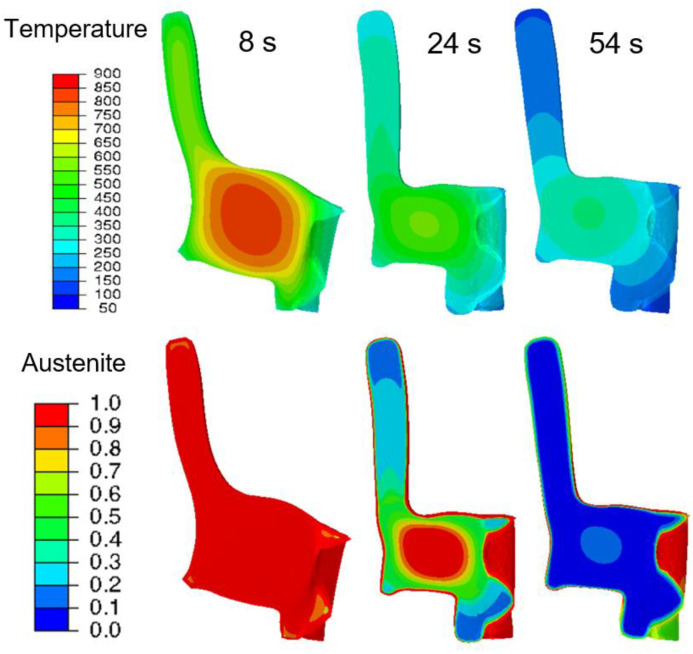
Contour plots and distribution of the temperature and austenite fractions at different quench times.

The first maximum or minimum was reached at around 7 s for all the tilt angles. From the simulation contour plot (Figure 14 (right)), it can be seen that the gear tooth and the lower part of the inside of the gear rim cooled faster than the web and hub area. Additionally, until 7 s, no phase transformation occured, so the tilting was caused purely by thermal strain. After 7 s (Figure 15), the further temperature development and the beginning of the phase transformation at the gear rim and upper and lower part of the hub changed the tilting behavior at all areas and was in the opposite direction, and reached the second extrema at around 24 s. With further cooling, the tilt development was dominated purely by phase transformation, and reached the third extrema at around 54 s. The transformation at the carburized layer then led to further changes in the tilt development at the gear tooth, gear rim, and the web area. The tilt angles at the end of the quenching were small and could be correlated with the heavy mass, which made the geometry more stable.

#### 3.3.2. Effect of Web Width

In this subsection, the effect of web width, i.e., the removal of material from the web area, is quantified by comparing the geometry types G1 and G2. The material removal was done in such a way that G2 has an almost perfect symmetric web position.

**Figure 16:**
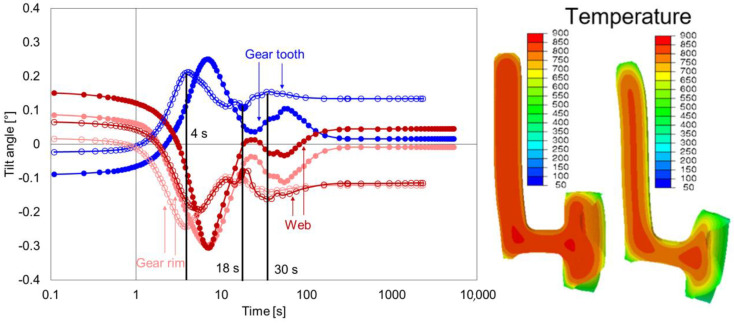
Tilt angle development of the web, gear rim, and gear tooth for the geometry type G2 (circle without fill) and comparison with geometry type G1 (circle with fill) during quenching (**left**), and temperature distribution and contour plots for G2 after 2 s and 4 s (**right**).

Figure 16 shows the comparison of tilt development of the gear tooth, gear rim, and web of geometry types G1 and G2. The removal of material at the web area led to significant changes in tilt behavior for all areas, which started during heating. The tilting was reduced after heating for the geometry G2 compared to G1. During quenching, the first extremum in the case of G2 occured earlier, i.e., reached around 4 s for the gear rim and gear tooth. The tilting of these areas was primarily caused by the asymmetrical radial shrinkage behavior of the upper and lower part of the gear rim. The tilting of the web proceeded similarly but reached the first extreme value after a delay of 6 s. This behavior can be explained by the effect of faster cooling at the lower part of the hub and gear rim on the web. In both cases, large radial temperature gradients resulted at the bottom of the web, leading to faster cooling of the bottom side of the web compared to the upper side of the web. With further cooling (18 s), the phase transformation at the hub and web area led to further changes in the tilt development. The occurrence of the event was the same as in geometry G1, but at different time points. Additionally, due to the reduced weight of G2, the stiffness was reduced, which increased the tilt angles at the end compared to geometry type G1.

#### 3.3.3. Thick Hub and Effect of Web Position

**Figure 17:**
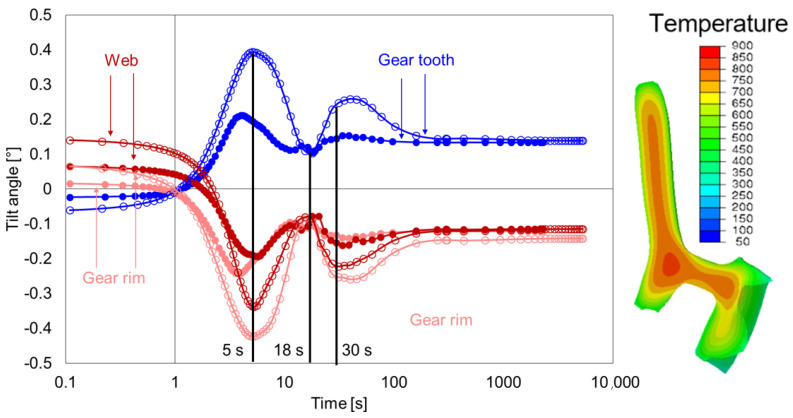
Tilt angle development of the web, gear rim, and gear tooth for the geometry type G3 (circle without fill) and comparison with geometry type G2 (circle with fill) during quenching (**left**), and temperature distribution and contour plot for G3 after 5 s (**right**).

In this subsection, the geometry types G2 and G3 are compared, and the effect of web position on the distortion behavior is analyzed. The web in G3 was shifted upwards to the maximum possible position, and with that, a maximum asymmetry of web position was achieved. The tilt development of the gear tooth, gear rim, and web of geometry type G2 and G3 are compared in Figure 17.

The effect of asymmetric web position on the distortion was clearly noticeable directly after heating. The tilt angles were increased for geometry G3 as compared to G2. The first extremum in the case of geometry G3 reached around 5 s, later compared to G2. The change in the tilt direction after 5 s was due to temperature development at the lower part of the hub, Figure 17, right. In G2, it was the upper gear rim area that cooled faster and affected the tilting behavior. In G3, however, due to the shift of web position upwards and unavailable space for radial shrinkage, instead of the upper gear rim area, the lower hub area affected the tilting. As the cooling advanced, the phase transformation at the hub and web areas of the gear dominated the titling development. Then, the transformation in the carburized layer at around 30 s changed the tilting behavior further. Surprisingly, at the end of the quenching, the tilting at the gear tooth and web for both geometry types was almost identical. Therefore, it seems that the effect of web position on the final distortion behavior at gear tooth and web is minimal for the given condition. However, the web position influences the tilting of the gear rim. The increase in asymmetry increased the tilt angle, i.e., the gear rim tilting in G3 larger than in G2.

#### 3.3.4. Asymmetric Web Position and Effect of Hub Width

**Figure 18:**
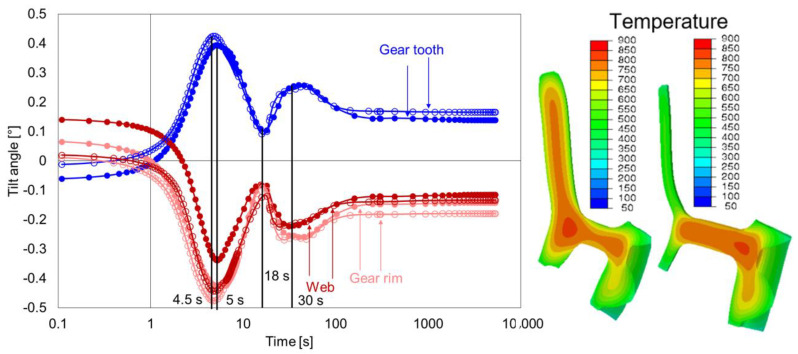
Tilt angle development of web, gear rim, and gear tooth for the geometry type G3 (circle with fill) and comparison with geometry type G5 (circle without fill) during quenching (**left**), and temperature distribution and contour plot for G3 and G5 after 5 s (**right**).

In this subsection, the effect of hub width is analyzed by comparing the geometry types G3 and G5 (Figure 18), where geometry type G5 had a thinner hub width.

The geometry G5, in comparison to G3, started with almost zero tilt angles for all the areas. The first extremum in the case of geometry G5 reached around 4.5 s, slightly earlier than G3 (5 s). In both cases, the change in the tilt direction after 5 s was due to temperature development at the lower part of the hub (Figure 18, right). The shift of the web position restricted the radial shrinkage at the gear rim, and the faster cooling at the hub exerted a moment on the web that influenced the tilting. Additionally, as the cooling advanced, the phase transformation at all areas of the gear dominated the titling development. Due to further mass reduction at the hub area, the overall geometry stiffness was further reduced. This resulted in an increase in tilt angles for G5 as compared to those of geometry type G3. Therefore, for the given condition, it can be assumed that the dimension of the hub influences the overall tilting behavior of the gear with asymmetric web position.

#### 3.3.5. Symmetric Web Position and Effect of Hub Width

**Figure 19:**
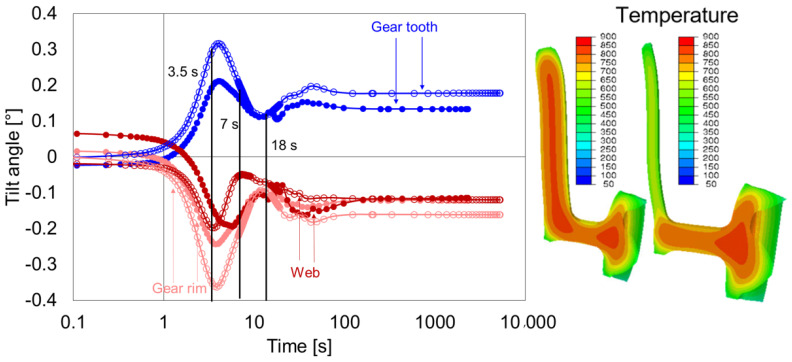
Tilt angle development of the web, gear rim, and gear tooth for the geometry type G4 (circle without fill) and comparison with geometry type G2 (circle with fill) during quenching (**left**), and temperature distribution and contour plot for G2 and G4 after 4 s (**right**).

The geometry types G2 and G4 were analyzed to quantify the effect of hub width on the geometry with a symmetric web. The tilt development of the gear tooth, gear rim, and web of geometry types G2 and G4 are compared in Figure 19.

The effect of hub width on the distortion behavior of the geometry with symmetric web position was noticeable directly after heating, specifically on the web area. The tilt angle of the gear rim and gear tooth for both geometries was more or less similar after heating, whereas the tilt angle for the web area for G2 was larger than G4. The first extremum in the case of geometry G4 reached around 3.5 s at the web, whereas the first extremum for G2 reached at 4 s at the gear tooth. The reason could be the temperature development at the hub and web areas. Due to the thin hub for G4, the hub cooled faster than the gear rim and affected the tilting development, Figure 19, right. Furthermore, the length of the web area for G4 was increased, which increased the radial shrinkage at the gear rim. The further advancement in the tilt development was due to phase transformation in all the areas. In this case too, it is assumed that the hub width had a significant effect on the distortion behavior and the material removal at the hub area increased the tilt angles. Therefore, independent of the web position, it can be said that the hub width has a strong influence on the overall distortion behavior.

#### 3.3.6. Thin Hub and Effect of Web Position (G4 and G5)

**Figure 20:**
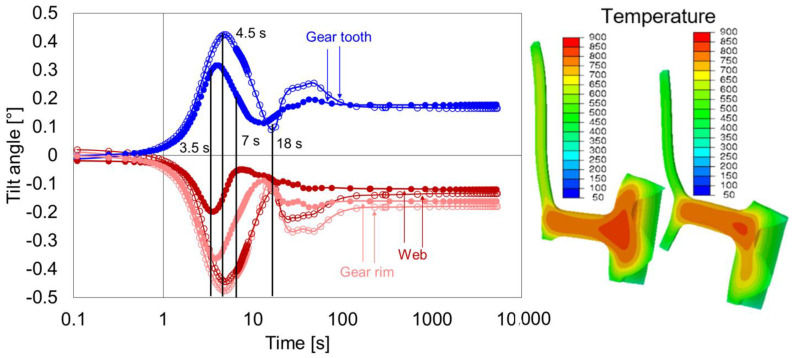
Tilt angle development of the web, gear rim, and gear tooth for the geometry type G4 (circle with fill) and comparison with geometry type G5 (circle without fill) during quenching (**left**), and temperature distribution and contour plot for G4 and G5 after 3.5 s (**right**).

In this subsection, the geometry types G4 and G5 are compared, and the effect of web position on the distortion behavior on the geometries with thin hub is analyzed. The web in G5 was at the possible maximum top position, and with that, a maximum asymmetry of web position was achieved. The tilt development of the gear tooth, gear rim, and web of geometry type G4 and G5 are compared in Figure 20.

For both the geometries, the tilt angle after heating was small, and till around 2 s of quenching time, the tilting development was perfectly identical. The first extremum in the case of geometry G4, as shown earlier, reached around 3.5 s at the web, and had a delay at the gear tooth and gear rim areas. In the case of G5, however, due to the shift of web position upwards and slower cooling at the upper gear rim area (Figure 20, right), the tilting development in the first phase was longer, and the first extremum reached around 4.5 s. The phase transformation dominated all further tilting development at all areas of the gear and the transformation in the carburized layer. At the end of the quenching, the tilt angles for both geometry types were almost similar. Therefore, for the given condition, it can be said that the effect of web position on the final distortion behavior is minimal for the geometries with thin hub width.

#### 3.3.7. Summarizing the Tilting Behavior of All Geometry Types

In the previous section, the tilt development during quenching for all the geometry types is shown and the effect of geometry variations is discussed. For all geometries, the tilt angles reached a maximum/minimum value depending on the occurrence of the events. The time to reach these maxima/minima depended strongly on the type of geometry. Table 3 and Table 4 show maxima/minima of the tilt angles of the gear tooth and web occurring during the complete quench period for geometry types G1–G5, respectively.

The tilting of the gear tooth and web ran exactly parallel for the geometry types G1, G3, and G5. The geometry G1 had a maximum possible width of the hub and gear rim, making the upper web and gear rim/gear tooth surfaces one single surface. The gear tooth and lower gear rim cool faster than the hub and web. Therefore, the tilting of the gear tooth downwards would also tilt the web and gear rim downwards with it. Nevertheless, it could also be the other way round, i.e., the web tilts and took the gear tooth with it. The geometry types G3 and G5 had the maximum possible upmost web position. The distance of the web position to the gear rim was so small that, here also, the tilting of gear tooth and web ran parallel, i.e., they had the same occurrence of the event as G1, but at different quench times.

In the cases of G2 and G4, the web position was almost symmetric to the gear rim. Furthermore, due to the thick hub of G2, the cooling was slower at the hub and web compared to the gear tooth and gear rim. Therefore, the first maximum was reached at the gear tooth, and then, after a slight delay, at the web. For G4, due to the thin hub, the cooling was faster at the hub, which exerts a moment on the web, and the maxima was first reached at the web, and then at the gear tooth. Due to symmetric web position, the tilt development at the web and gear tooth did not run parallel in either case.

The occurrence of different events and their influence on the tilting of gear tooth and web was almost similar, with a change in timing. However, the area at which the first maxima/minima occured strongly depended on the geometrical dimensions. In all cases, the tilting was first influenced by the distribution of thermal strains followed by the combination of thermal and transformation strains. These highly complex interactions of geometry-, thermal-, and transformation-related strains made it difficult and, in some cases, even impossible to understand whether the gear tooth, or web, or interplay between both was responsible for such tilting behavior.

## 4. Summary and Conclusions

In this paper, a 3D-FEM simulation of the case hardening process for different weight reduced counter gears was performed. With adjusted heat transfer coefficient and material parameters, the developed 3D simulation model predicted good and, in some cases, satisfactory shape and size change results compared to the experimental findings. The tilting behavior at different gear areas for all the geometry types was analyzed to understand the complex distortion behavior and correlate it with design modifications.

The simulation study has shown that the tilting of the gear tooth in the radial direction and the tilting of the web and gear rim in the axial direction result from the interplay of the different asymmetries of mass and temperature distribution. As expected, mass distribution plays a central role, as temperature development depends strongly on it. Depending on the dimensions of hub width and the web position, these areas contributed at different points during quenching period, and affected the distortion behavior.

The removal of material from the web area directly influenced the distortion behavior. Due to reduced weight, the stiffness was also reduced, which increased the tilt angles compared to the basic geometry. Furthermore, the effect of web position on the final tilting behavior was minimal, independent of the hub width. However, the geometries with similar web position but different hub width strongly affected distortion behavior. The reduction in the mass at the hub area further reduced the stiffness of the geometry, which increased the tilt angles of the gear.

The main findings of the paper are:

- The heat transfer coefficient is strongly position- and temperature-dependent, and, unexpectedly, also strongly geometry-dependent. It is therefore necessary for each geometry variation to preferably determine the HTC from cooling curve measurements. Alternatively, indirect fitting of HTC to the measured distortions must be performed. Asymmetries of mass and temperature distribution directly affect the distortion behavior.
- The tilting of the gear tooth in the radial direction can be approximated using a parabolic function. The parabolic approach allows for a separation of effects such as mean radius change, tilting, and convexity. The mean radius change and pure tilt are maintainable in manufacturing; however, convexity is difficult to handle, and therefore must be avoided.
- A linear approximation is sufficient to describe tilting in the axial direction for the investigated geometry types.
- The lighter the geometry, the higher the distortion induced in the gear.

Based on these results, future work will focus on compensation measures and study the influence of the optimization of cross-sectional areas of the gears on the distortion behavior. The cross-sectional radius can be either increased or designed using the method of tension triangles, which reduces notch effects at the cross-sectional area [26,27,28].

## Figures and Tables

**Table 1:** Chemical composition of the melt used (all results given in mass percent (ma.%)).

	C	Si	Mn	P	S	Cr	Mo	Ni	Al	Cu	N
Melt	0.21	0.1	1.31	0.011	0.023	1.13	0.06	0.18	0.032	0.13	0.009
Standard	0.17–0.22	≤0.4	1.10–1.40	≤0.035	≤0.035	1.00–1.30	-	-	-	-	-

**Table 2:** Used local heat transfer descriptions.

Geometry	Hub	Web Top	Web Bottom	Gear Rim	Toothing
G2	Hub G1	Web G1	Web G1	Gear rim G1	Toothing G4
G3	Hub G1	Web G1	Modified	Modified	Toothing G4
G5	Hub G4	Web G4	Modified	Modified	Toothing G4

**Table 3:** Time to reach maximum/minimum tilting of the gear tooth for all geometry types.

	First Max/Min	Second Max/Min	Third Max/Min
G1	7 s	24 s	54 s
G2	4 s	18 s	40 s
G3	5 s	18 s	40 s
G4	4 s	13 s	36 s
G5	4.5 s	18 s	36 s

**Table 4:** Time to reach maximum/minimum tilting of the web for all geometry types.

	First Max/Min	Second Max/Min	Third Max/Min
G1	7 s	24 s	54 s
G2	6 s	18 s	40 s
G3	5 s	18 s	40 s
G4	3.5 s	7 s	36 s
G5	4.5 s	18 s	36 s

## Data Availability

The data presented in this study are available on request from the corresponding author.
